# Hide and Seek: How Lyme Disease Spirochetes Overcome Complement Attack

**DOI:** 10.3389/fimmu.2016.00385

**Published:** 2016-09-26

**Authors:** Peter Kraiczy

**Affiliations:** ^1^Institute of Medical Microbiology and Infection Control, University Hospital of Frankfurt, Frankfurt am Main, Germany

**Keywords:** spirochetes, *Borrelia*, Lyme disease, complement, immune evasion, complement regulator, innate immunity

## Abstract

Overcoming the first line of the innate immune system is a general hallmark of pathogenic microbes to avoid recognition and to enter the human host. In particular, spirochetes belonging to the *Borrelia burgdorferi* sensu lato complex have developed various means to counter the immune response and to successfully survive in diverse host environments for a prolonged period of time. In regard to complement resistance, *Borrelia* utilize a plethora of immune evasion strategies involves capturing of host-derived complement regulators, terminating complement activation as well as shedding of cell-destroying complement complexes to manipulate and to expeditiously inhibit human complement. Owing to their mode of action, the interacting surface-exposed proteins identified among *B. burgdorferi* sensu stricto (s.s.), *Borrelia afzelii, Borrelia spielmanii*, and *Borrelia bavariensis* can be classified into at least two major categories, namely, molecules that directly interfere with distinct complement components including BBK32, CspA, BGA66, BGA71, and a CD59-like protein or molecules, which indirectly counteract complement activation by binding various complement regulators such as Factor H, Factor H-like protein 1 (FHL-1), Factor H-related proteins FHR-1, FHR-2, or C4Bp. The latter group of genetically and structurally unrelated proteins has been collectively referred to as “complement regulator-acquiring surface proteins” and consists of CspA, CspZ, ErpA, ErpC, ErpP, and the as yet unidentified protein p43. This review focuses on the current knowledge of immune evasion mechanisms exhibited by Lyme disease spirochetes and highlights the role of complement-interfering, infection-associated molecules playing an important part in these processes. Deciphering the immune evasion strategies may provide novel avenues for improved diagnostic approaches and therapeutic interventions.

## Introduction

The genus *Borrelia* (*B*.) comprises the causative agents of Lyme disease (LD) and relapsing fever (1–3). Concerning LD spirochetes, there are distinct species belonging to the *Borrelia burgdorferi* sensu lato complex of which six species including *B. burgdorferi* sensu stricto (s.s.), *Borrelia afzelii, Borrelia garinii, Borrelia spielmanii, Borrelia bavariensis* (formerly referred to as *B. garinii* OspA serotype 4), as well as candidatus *Borrelia mayonii*, are associated with human LD (4, 5). While *Borrelia valaisiana, Borrelia lusitaniae*, and *Borrelia bissettii* have been detected in human biopsies, the pathogenicity of these and the remaining borrelial species remains largely unclear (6–8).

To survive and establish a persistent infection in the human host, pathogens must evade the first line of host defense by counteracting complement as an essential part of innate immunity. This powerful surveillance system comprises a network of precursors, regulatory and inhibitory proteins that can be immediately activated upon recognition of invading microorganisms (9, 10). Despite the effectiveness and abundance of complement, LD spirochetes are able to overcome its destructive defense mechanisms (11–13). While attempting to decipher the molecular mechanisms of complement evasion, distinct complement-interfering and -inhibiting molecules of *B. burgdorferi* s.s., *B. afzelii, B. spielmanii*, and *B. bavariensis* have been identified (14, 15). This review focuses on the current knowledge of the molecular principles utilized by LD spirochetes to counteract complement at certain activation levels and on the borrelial proteins known to take part in complement inactivation.

## Activation and Regulation of the Complement System

Complement operates as a cooperative network of inactive precursor molecules, fluid-phase and membrane-bound regulators, and inhibitors (16–20). The initiation of complement takes place in a cascade-like manner through three activation routes: the classical (CP), the lectin (LP), and the alternative pathway (AP), all of which converge in the generation of the highly reactive molecule, C3b (17, 19, 21, 22). The CP can be activated after binding of C1q to immune complexes (IgM and IgG) or charged molecules on the bacterial surface (23). In complex with C1q and C1r, C1s mediates cleavage of C4 and C2 leading to the formation of the C3 convertase, C4b2b. Activation of the LP is initiated by binding of mannan-binding lectin (MBL), ficolins (H-ficolin, L-ficolin, and M-ficolin), or collectins associated with MBL-associated serine proteases (MASP), to carbohydrates of microbial origin. After activation of MASP-2 by MASP-1, both proteases cleave C2 while MASP-2 is able to also cleave C4 to generate the identical C3 convertase. Finally, the AP is initiated by spontaneous hydrolysis of C3 followed by binding of C3b (opsonization) to different targets on the bacterial surface. Recruitment of Factor B (FB) followed by Factor D (FD)-mediated cleavage results in the formation of the membrane-bound C3 convertase C3bBb. To extend the half-life and to trigger the amplification of C3b (feedback loop), the C3 convertases of the AP are stabilized by properdin. Of note, deposition of large quantities of C3b on microbial surfaces is a prerequisite for opsonization and phagocytosis of invading pathogens. Upon binding of newly generated C3b molecules, the C4b2b and C3Bb complexes serve as precursors for the C5 convertases C4b2b3b and C3bBb3b. Cleavage of C5 into C5a and C5b by the C5 convertases initiates the unidirectional, sequential binding of the late components C6, C7, and C8 to C5b. Once the C5b–8 complex is formed, polymerization of multiple C9 molecules ensues, finally generating the pore-forming terminal complement complex [C5b–9, TCC, also referred to as membrane attack complex (MAC)], which promotes lysis of susceptible cells (19, 20). To avoid the detrimental effects of excessive complement activation, this surveillance system is tightly controlled by soluble and membrane-anchored regulators (21). The soluble regulators of the CP and LP include C1 esterase inhibitor (C1-INH) and C4b-binding protein (C4Bp), while the AP is primarily regulated by Factor H (FH) and Factor H-like protein 1 (FHL-1). Vitronectin, clusterin, and, in part, FH-related protein 1 (FHR-1) comprise the regulatory proteins of the terminal activation sequence (21, 24).

## Diversity in Complement Susceptibility

Initial investigations showed that LD spirochetes differ substantially in their susceptibility to human serum and finally led to the classification of spirochetes into three main categories, serum-resistant, intermediately serum-resistant/sensitive or partially resistant, and serum-sensitive (25–27). It is worth mentioning that categorizing of spirochetes in these particular groups largely depends on technical parameters, e.g., serum collection and storage, serum and cell concentrations, incubation period, and the method of choice used to determine borrelial survival, making it difficult to compare the data published. Changing the experimental conditions can lead to differences in the phenotypic classification, in particular of intermediately serum-resistant/sensitive strains. Among LD spirochetes, *B. burgdorferi* s.s., *B. afzelii, B. spielmanii, B. bavariensis*, and *Borrelia japonica* are resistant to complement-mediated killing, *B. bissettii* was classified as intermediately serum-resistant and *B. garinii, B. valaisiana*, and *B. lusitaniae* comprise the group of highly susceptible spirochetes (13, 25–35). Furthermore, differences in serum susceptibility have been reported among certain *B. valaisiana* and *B. garinii* isolates (29, 36). Strikingly, the serum susceptibility pattern of LD spirochetes almost matches pathogenicity in humans with the exception of *B. garinii* known to frequently cause LD. The underlying molecular principles of how *B. garinii* circumvent complement-mediated killing are largely unknown and are still a matter of controversy. It is likely that pathogen-associated factors produced solely in the infected host or that interaction with host or tick-derived proteins upon the transmission process, e.g., plasminogen (37), Tick Salivary Lectin Pathway Inhibitor (TSLPI) (38), or Salp20 (39), may protect *B. garinii* from complement attack.

## Borrelial Proteins Interacting with Human Complement Regulators

### Inactivation of the Alternative Pathway by Binding Complement Regulators FH and FHL-1

**Figure 1:**
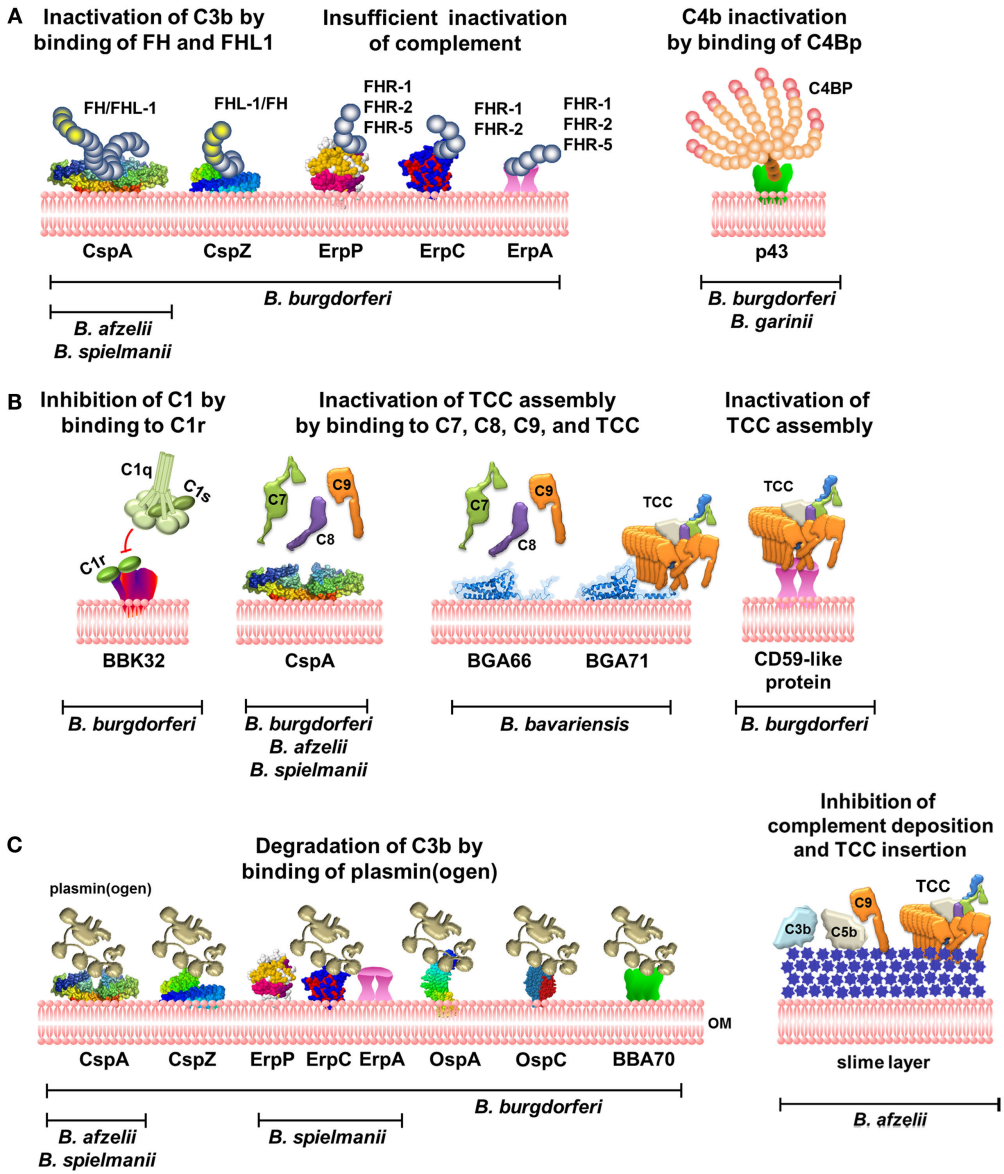
**Complement evasion strategies of LD spirochetes**. **(A)** Inhibition of the AP and CP by binding of complement regulators FH and FHL-1 to CspA and CspZ or C4Bp to p43. Binding of FHRs to ErpP, ErpC, and ErpA does not terminate complement activation. **(B)** Inhibition of the CP and TP by direct interaction of diverse borrelial proteins produced by distinct genospecies with C1r or late complement components. **(C)** Inactivation of C3b by binding of plasmin(ogen) by diverse borrelial proteins and prevention of complement deposition by the production of a mucoid layer. OM, outer membrane; TCC, terminal complement complex; FH, Factor H; C4Bp, C4b-binding protein.

In 1997, two independent studies demonstrated that serum-resistant strains exhibit significantly lower amounts of deposited activation products (C3, C6, and TCC) compared to serum-susceptible strains, leading to the assumption that the complement cascade is inhibited at the level of C3 and/or C5 activation; however, no underlying mechanism(s) were elucidated (26, 27). Several years later in 2001, OspE of *B. burgdorferi* s.s. (40) and the so-called Complement Regulator-Acquiring Surface Proteins (CRASP) of *B. burgdorferi* s.s. and *B. afzelii* (41, 42) were identified as ligands for FH and, in part, for FHL-1. Binding of these complement regulators by serum-resistant spirochetes inhibits activation at the central step of the complement cascade, C3 activation and the formation of C3 convertase, and thereby terminates the assembly and finally the integration of the TCC into the bacterial membrane (28, 42) (Figure 1A). Thereafter, several FH-binding proteins were detected in serum-resistant *B. spielmanii, B. japonica*, and *B. bissettii* isolates (31, 43, 44), while serum-sensitive *B. garinii, B. lusitaniae*, and *B. valaisiana* isolates did not bind functionally active FH (28, 30, 36, 42). The importance of disrupting complement activation at the level of C3 by surface-bound FH and FHL-1 was confirmed both in initial and follow-up studies investigating different borrelial species (29, 31–33). With the exception of serum-resistant *B. bavariensis* (45), almost all serum-resistant borrelial species are able to co-opt human FH and FHL-1 to protect themselves from complement-mediated killing, allowing LD spirochetes to survive in humans and in diverse, immune competent animal hosts (13).

Concerning the FH/FHL-1/FHR interacting molecules, up to five distinct outer surface lipoproteins, collectively termed CRASP, have been identified, comprising three genetically unrelated groups with partially overlapping biological functions (Table 1) (14, 15, 41). Owing to the genetic composition, different combinations of these proteins can be exposed at the surface of a particular isolate. For historical reasons, a variety of names have been introduced at the time of description and are still found in the literature, leading to considerable confusion about their identities and biological functions. As a simplification, the synonyms and additional designations of the protein and gene names of CRASPs along with their specific functional properties are summarized in Table 1. With regard to the reference type strain *B. burgdorferi* s.s. B31, the identified FH/FHL-1/FHR-binding proteins consists of CspA (CRASP-1, BBA68), CspZ (CRASP-2, BBH06), ErpP (CRASP-3, BBN38), ErpC (CRASP-4), and ErpA (CRASP-5, BBP38) (14). Due to their different functions, structures, gene organization, etc., OspE homologous proteins are collectively referred to as OspE-related proteins (Erp) proteins (46).

**Table 1:** **Characteristics of complement-interacting proteins of LD spirochetes**.

	CspA	CspZ	ErpP^a^	ErpC^a^	ErpA^a^	p43	BBK32	BGA66	BGA71	CD59-like protein
Synonyms and other designations	CRASP-1	CRASP-2	CRASP-3	CRASP-4	CRASP-5	–	–	–	–	–
	BbCRASP-1	BbCRASP-2	BbCRASP-3 BBN38	BbCRASP-4	BbCRASP-5					
	BBA68	BBH06			ErpI					
	ZS7.A68				ErpN					
	FHBP				BBP38					
					BBL39					
					OspE					
Gene name	*cspA*	*cspZ*	*erpP*	*erpC*	*erpA*	ND	*bbk32*	*bga66*	*bga71*	ND
Origin	Bb, Ba, Bs	Bb	Bb	Bb	Bb	Bb	Bb	Bba	Bba	Bb
Confers serum resistance	Yes	Yes	No	No	No	ND	Yes	Yes	Yes	ND
Interaction with complement regulators/components	FH	FH	FHR-1	FHR-1	FHR-1	C4Bp	C1r	C7, C8, C9, TCC	C7, C8, C9, TCC	TCC
	FHL-1	FHL-1	FHR-2	FHR-2	FHR-2					
	C7, C8, C9, TCC		FHR-5		FHR-5					
Interaction with plasmin(ogen)	Yes	Yes	Yes	Yes	Yes	ND	ND	ND	ND	ND
Affected complement pathways	AP, TP	AP	–	–	–	CP/LP(?)	CP	TP	TP	TP

*^a^Binding of FH has only been confirmed for recombinant proteins*.

*ND, not determined; CRASP, complement regulator-acquiring surface protein; Erp, OspE/F-like protein; FH, Factor H; FHL, Factor H-like protein, FHR, FH-related protein; TCC, terminal complement complex; Bb, B. burgdorferi; Bba, B. bavariensis; Ba, B. afzelii; Bs, B. spielmanii; AP, alternative pathway; CP, classical pathway; LP, lectin pathway; TP, terminal pathway*.

*CspA* is the predominant FH and FHL-1 binding protein of *B. burgdorferi* s.s. and belongs to the paralogous protein family PFam54, of which 11 paralogs are produced in strain B31. Except for CspA, none of the other PFam54 members interact with FH and FHL-1, despite the high sequence homology, suggesting that these proteins possess other, as yet unknown functions (47). Moreover, irrespective of geographical origin, CspA paralogs among *B. burgdorferi* s.s. isolates are highly conserved (48). More importantly, structure refinements have disclosed a homodimer as the biologically relevant architecture of CspA (Figure 1A) (49). Although sequence differences within the C-terminal region may account for the inability of CspA paralogs to bind FH and FHL-1, further investigations are necessary to satisfactorily clarify this issue. Initial studies revealed a strong binding affinity of both complement regulators to CspA, accompanied by a powerful capacity to inactivate C3b in the presence of Factor I (50, 51) (Figure 1A). The importance of CspA in facilitating complement resistance of *B. burgdorferi* s.s. has been clearly demonstrated by generating a *cspA*-deficient mutant and strains complemented with the *cspA* gene (52–54). More recently, CspA has been demonstrated to possess additional functions: this protein directly interacts with components of the terminal pathway (C7, C8, and C9) as well as plasmin(ogen), thereby terminating TCC assembly and upon activation to plasmin also promoting degradation of C3b (54, 55) (Figures 1B,C). CspA orthologs, sharing identical biological functions, were also identified in *B. afzelii* and *B. spielmanii*. All of these orthologs belong to the PFam54 protein family, but the loci of the encoding genes differ from *cspA* of *B. burgdorferi* s.s. The orthologs display the same inactivating properties as CspA, impart resistance to complement-mediated killing, and bind complement components FH, FHL-1, C7, C8, and C9 as well as plasmin(ogen) (43, 55–57). These findings suggest that CspA is an important serum resistance factor of *B. burgdorferi* s.s., *B. afzelii*, and *B. spielmanii*. CspA is produced during tick feeding, shortly after transmission to the mammalian host and during transmission to feeding, naïve ticks but not in the midgut of unfed ticks, suggesting that CspA protect spirochetes from complement attack during established infection (58, 59).

*Borrelia burgdorferi* s.s. produces an additional FH and FHL-1-binding protein, *CspZ*, which independently provides borrelial cells with resistance to human complement (60, 61) (Figure 1A; Table 1). Once FH or FHL-1 binds to the CspZ-producing spirochetes, termination of the complement cascade takes place at the activation level of C3 as demonstrated by the decay of the C3 convertase, an increase of C3b degradation products, and the lack of deposited TCC (60, 61). Although *cspZ* sequences were identified in numerous genospecies associated with LD, including *B. afzelii, B. garinii, B. spielmanii, B. bavariensis*, and *B. bissettii*, none of the other CspZ proteins interact with FH and FHL-1 (62–65). Although CspZ is produced during mammalian infection and elicits a robust antibody response (66), additional studies revealed that this protein does not protect mice from infections and, if at all, is only partially required for infection (63, 67, 68). In addition, CspZ like CspA has been identified as a plasmin(ogen)-binding molecule, enabling *B. burgdorferi* s.s. to degrade surface-bound C3 and C3b (57) (Figure 1C). Of note, the efficacy in degrading C3/C3b by plasmin is much less pronounced, compared to the C3b inactivation capacity of FH and FHL-1 (55). In the case of strong complement activation initiated by the AP, generation of high amounts of C3b cannot be sufficiently inactivated by surface-bound plasmin; thus, more adequate inhibitors such as FH or FHL-1 are required to overcome the feedback loop.

Lyme disease spirochetes produce a number of polymorphic proteins belonging to the OspE/F paralogous protein family PFam162, of which *ErpA (BBP38), ErpC*, and *ErpP (BBN38)* have been reported to bind FH as well as FHR-1, FHR-2, and in part FHR-5 (40, 50, 69–74) (Figure 1A; Table 1). Despite binding of FH to purified Erp proteins, there are several lines of evidence indicating that the same molecules, when exposed to the bacterial surface, do not confer protection of LD spirochetes from deposition of C3 and TCC (14, 70, 74). Spirochetes producing Erp proteins, but lacking CspA and CspZ, display a susceptible phenotype and are readily killed by complement (52, 70, 74). Owing to the Erp proteins strong affinity for FHRs (50), FH might be displaced from the bacterial surface, with a concurrent loss of its complement regulatory functions and, as such, is unable to protect the cells from the deleterious effects of complement.

Besides binding of complement components, ErpA, ErpC, and ErpP, as well as other Erp orthologs, are known to serve as potential ligands for plasmin(ogen) (Figure 1C) (75, 76). As mentioned previously, the role of activated plasmin in complement evasion of LD spirochetes requires further investigations. Although additional FH-binding Erp orthologs were identified in *in vitro* cultivated *B. garinii, Borrelia andersonii, B. japonica, Borrelia turdi*, and *Borrelia tanukii* isolates (44, 72, 77), the impact of these molecules on complement resistance has never been confirmed, in particular Erp proteins of *B. garinii*. Concerning additional FH/FHL-1-binding proteins, no data are currently available on other LD *Borrelia* species.

### Inactivation of the Classical Pathway by Binding Complement Regulator C4Bp

The role of C4Bp, the key regulator of the CP, in immune evasion of LD spirochetes is still a matter of controversy. Pietikainen et al. have observed binding of C4Bp in serum-resistant *B. burgdorferi* s.s. and *B. afzelii* as well as in serum-sensitive *B. garinii* isolates (78) (Figure 1A). C4Bp bound to the borrelial surface in concert with Factor I maintained its complement regulatory activity and inactivated C4b (78). Along with the determination of C4Bp binding, a 43-kDa protein, tentatively designated *p43* was identified in *B. burgdorferi* strains B31 and N40, and *B. garinii* strains g46 and g50 (78). However, other studies failed to show C4Bp binding by *B. burgdorferi* s.s. LW2, *B. garinii* G1, *B. valaisiana* (*n* = 3), and *B. bavariensis* strains (*n* = 8) (36, 45), possibly due to different techniques or antibodies used for the detection of C4Bp. In addition, taking into consideration that *B. garinii* cells are killed in serum concentrations >20%, the physiological relevance of C4Bp in promoting complement resistance remains to be determined.

## Borrelial Proteins Displaying Complement-Inhibitory Activity

More recently, a novel immune evasion mechanism has been described by which *B. burgdorferi* s.s. specifically blocks CP activation (79). This study depicts *BBK32* as the first protein that binds to C1r in a non-covalent manner and thereby preventing autocatalysis of this proenzyme and subsequently the cleavage of C1s, leaving the C1q complex in an inactive enzymatic state (Figure 1B; Table 1). By interfering with C1r, BBK32 acts as a potent inhibitor of the CP without affecting the LP and AP.

Unlike other serum-resistant LD spirochetes, *B. bavariensis* binds neither FH/FHL-1 nor other complement regulators such as C4Bp or C1-Inhibitor (45). Detailed analysis revealed two proteins, *BGA66* and *BGA71* as novel complement inhibitors. They belong to the PFam54 protein family and share 51 and 41% sequence identities, respectively, to CspA. Both proteins interact with components of the terminal pathway, in particular C7, C8, and C9, and also with the assembled TCC (45) (Figure 1B; Table 1). Binding of the borrelial proteins to various components of the terminal pathway affects TCC formation by (i) inhibiting C9 auto-polymerization, (ii) terminating TCC assembly, and (iii) preventing integration of the functional pore-forming complex. Moreover, BGA66 and BGA71 are simultaneously produced in all *B. bavariensis* strains investigated, but each protein by itself displays anti-complement activity and renders transformed spirochetes resistant to complement-mediated killing. Despite the structural similarities to CspA, BGA66 and BGA71 do not bind the potent complement regulators FH and FHL-1 (45). One might speculate that termination of the final activation steps may result in a somewhat weaker complement inactivation capacity. However, this does not appear to be the case as CspA and CspZ-producing and FH/FHL-1-binding spirochetes did not show a different resistance phenotype compared to BGA66- or BGA71-producing cells, indicating that inhibition of the terminal pathway is as efficient as blocking complement activation at the level of C3.

*Borrelia burgdorferi* s.s. also produces a CD59-like protein, which preferentially bind to C9 and to some extent to the β-subunit of C8 (80) (Figure 1B; Table 1). By using anti-CD59 antibodies, the functional activity of this protein could be blocked, rendering the spirochetes susceptible to complement-mediated killing. Although this surface-exposed protein has never been identified, the binding properties of the CD59-like protein suggest a role in inactivating the terminal pathway of complement. Despite the overlapping complement-inhibitory activities, the CD59-like protein is not identical with BGA66 and BGA71.

## Further Proteins and Structures Involved in Complement Resistance of LD Spirochetes

Besides the already mentioned molecules, which interacting with complement in multiple ways, additional proteins have been described that also bind human plasmin(ogen), e.g., OspA, OspC, and BBA70 (81–83) (Figure 1C). For the latter, degradation of C3 and, in part, C5 has been demonstrated. Whether the interactions of OspC and BBA70, known to be expressed in the mammalian host, might support complement inactivation *in vivo* is not known.

Initial studies of the molecular principles of complement resistance, focusing on the amounts of deposited complement components, revealed an amorphous structure of high density that surrounds the entire cell envelope and, apparently, acts as a physical barrier, preventing the insertion of the formed TCC into the bacterial membrane of serum-resistant cells (Figure 1C) (32). Currently, no data are available on the composition and content of this so-called “slime layer” and whether this structure is present in LD spirochetes other than *B. afzelii* (32). Thus, further studies are required to verify the precise nature of this morphological substance.

## Future Directions

Over the last decades, numerous molecules have been identified that interact with the innate immune system in multiple ways to influence or terminate complement at distinct activation levels, e.g., initiation of the CP, C3 activation by the AP, and formation of the TCC. Knowledge of the proteins involved in the interaction with complement has allowed for a better understanding of molecular principles of complement evasion developed by LD spirochetes. Future investigations will undoubtedly identify additional complement-interacting molecules required for evading the innate immune response of different animals, including reservoir hosts, and provide insight into whether these proteins might function in a host-specific manner during the infection process.

## Author Contributions

PK prepared the figure and table and wrote the manuscript.

## Conflict of Interest Statement

The author declares that the research was conducted in the absence of any commercial or financial relationships that could be construed as a potential conflict of interest.
